# Structural and Luminescence Properties of Lu_2_O_3_:Eu^3+^ F127 Tri-Block Copolymer Modified Thin Films Prepared by Sol-Gel Method

**DOI:** 10.3390/ma6030713

**Published:** 2013-02-26

**Authors:** Angel de Jesus Morales Ramírez, Margarita García Hernández, Antonieta García Murillo, Felipe de Jesús Carrillo Romo, Joel Moreno Palmerin, Dulce Yolotzin Medina Velazquez, María Luz Carrera Jota

**Affiliations:** 1 Instituto Politécnico Nacional, CIITEC IPN, Cerrada de Cecati S/N. Col. Santa Catarina, Azcapotzalco México D.F. C.P. 02250, Mexico; E-Mails: angarciam@ipn.mx (A.G.M.); fcarillo@ipn.mx (F.J.C.R.); mcarreraj1100@alumno.ipn.mx (M.L.C.J.); 2 Departamento de Ciencias Naturales, Universidad Autónoma Metropolitana, Unidad Cuajimalpa, DCNI, Pedro Antonio de los Santos 84, México D.F. 11850, Mexico; E-Mail: mgarciah@correo.cua.uam.mx; 3 CINVESTAV Querétaro, Libramiento Norponiente #2000, Fracc. Real de Juriquilla, Querétaro C.P. 76230, Mexico; E-Mail: jmoreno@qro.cinvestav.mx; 4 Ciencias Básicas e Ingeniería, Universidad Autónoma Metropolitana-Azcapotzalco, Av. San Pablo No 180, Col. Reynosa-Tamaulipas, México D.F. C.P. 02200, Mexico; E-Mail: dyolotzin@correo.azc.uam.mx

**Keywords:** Lu_2_O_3_, luminescence, m-lines, F127, tri-block copolymer

## Abstract

Lu_2_O_3_:Eu^3+^ transparent, high density, and optical quality thin films were prepared using the sol-gel dip-coating technique, starting with lutetium and europium nitrates as precursors and followed by hydrolysis in an ethanol-ethylene glycol solution. Acetic acid and acetylacetonate were incorporated in order to adjust pH and as a sol stabilizer. In order to increment the thickness of the films and orient the structure, F127 Pluronic acid was incorporated during the sol formation. Structural, morphological, and optical properties of the films were investigated for different F127/Lu molar ratios (0–5) in order to obtain high optical quality films with enhanced thickness compared with the traditional method. X-ray diffraction (XRD) shows that the films present a highly oriented cubic structure <111> beyond 1073 K for a 3-layer film, on silica glass substrates. The thickness, density, porosity, and refractive index evolution of the films were investigated by means of m-lines microscopy along with the morphology by scanning electron microscope (SEM) and luminescent properties.

## 1. Introduction

Eu^3+^ doped Lu_2_O_3_ phosphor has attracted attention in recent years due to its exceptional properties, such as: high density (9.4 g/cm^3^), high Z number, (Z = 71), nonhygroscopicity, high chemical and thermal stability, and high luminescent efficiency [1,2,3]. Furthermore, it is capable of effectively absorbing any type of ionizing radiation [4], which makes it very suitable for high-resolution X-ray imagining systems, including high definition X-ray radiographies, Positron Emission Tomography (PET) scanners, and many industrial measuring systems [5,6]. Furthermore, other applications like high resolution devices such as cathode-ray tubes (CRTs), field emission displays (FEDs) [7], solid state laser [8], or use in microelectronics [9] has also been proposed. In particular for luminescent applications, it is important to produce transparent thin-film phosphors since they present outstanding advantages such as high contrast and resolution, as well as better adhesion and better physical homogeneity [10,11]. Therefore, Lu_2_O_3_ has been prepared as a thin film by using several methods, including: Chemical vapor deposition (CVD) [12], physical vapor deposition (PVD) [13], pulsed laser deposition (PLD) [9], nanoparticles co-precipitation method and painting technique [14], hot-pressing [15,16], atomic layer deposition [17], and sol gel method [18,19,20]. The last one has emerged as one of the most promising processes as it is particularly efficient in producing transparent, homogeneous oxide layers on different substrates at relatively low cost [21]. The sol-gel method comprises of the deposition of films by dip or spin coating and the conversion of the xerogels films to ceramic thin films via heat treatment. However, it is difficult to achieve thick films without cracking along the ideal thickness in order to satisfy practical applications. A possible alternative is the incorporation of a high boiling point reagent into the sol such as the polyvinylpyrrolidone (PVP), which has been employed in luminescent systems such as Gd_2_O_3_:Eu^3+^ [22], GdTaO_4_:Eu^3+^ [23] and BaTiO_3_:Eu^3+^ [24]. A different alternative is to add Pluronic F127 acid, a PEO_106_PPO_70_PEO_106_ (PEO = polyethylene oxide, PPO = polypropylene oxide) tri-block copolymer with an average molecular weight of 13600 g mol^−1^, which has also been found to act as a structure-directing agent in TiO_2_ [25,26,27] in catalytic and solar cells applications. 

In this work, the structural, morphological and optical properties of transparent and crack-free Lu_2_O_3_:Eu^+3^ sol-gel derived thin films deposited on SiO_2_ glass by a sol-gel dip coating technique, adding the tri-block copolymer F127 into the sol, are reported. The structural evolution was evaluated by X-ray diffraction (XRD), and the morphological studies were achieved by means of a scanning electron microscope (SEM). Furthermore, the optogeometric parameters and the density and porosity of the dip-coated films were measured by m-lines spectroscopy, and tests were carried out in films with different F127/Lu molar ratio. Finally, the luminescent properties of the Lu_2_O_3_:Eu^3+^ F127 modified thin films were analyzed. 

## 2. Results and Discussion

### 2.1. Structural and Morphological Studies

**Figure 1:**
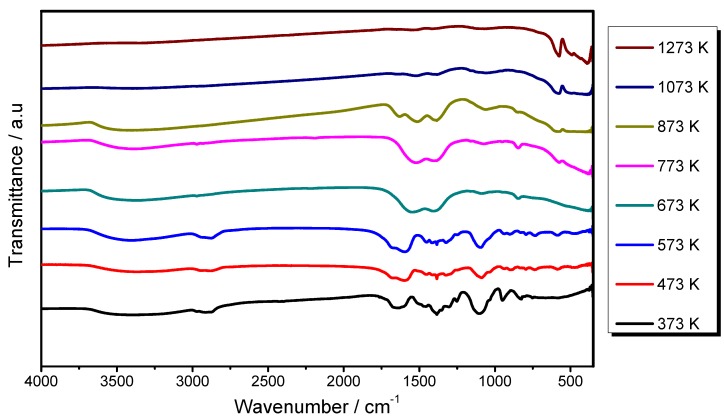
Fourier transform infrared (FTIR) spectra of Chemical evolution at Lu_2_O_3_:Eu^3+^ F127 modified system, F127/Lu = 2.0.

Figure 1 shows the Fourier transform infrared (FTIR) spectra of the chemical evolution of the sol-gel process for the synthesis of the Lu_2_O_3_:Eu^3+^ F127 modified thin film. The study was carried out for the F127/Lu = 2 sample, using the KBr pelleting technique for processed powders at different annealing temperatures. As can be observed for the xerogel dried at 373 K and 473 K, there is a strong presence of the O–H bands from the presence of water and alcohol groups (ethanol and etyleneglycol), characterized for the bands at 3300 cm^−1^ (ν), 1650 cm^−1^ (δ) and 750 cm^−1^ (δ), ascribed to O–H stretching (ν) and deformation (δ) vibrations. These bands are still present beyond 1073 K, indicating that they are still adsorbed into the surface of the sample. In addition, strong absorption bands are present at 1089 cm^−1^ and 850 cm^−1^, which can be attributed to a symmetrical stretching of C–O and deformation vibrations of C–O in CO_3_^2−^, respectively due to the thermal decomposition of the carbon groups of acetic acid and etyleneglycol. On the other hand, the absorption band localized at 1380 cm^−1^ is ascribed to a N–O stretching vibration [28] of NO_3_^−^. All of the bands observed at 1360 and 1500 cm^−1^ correspond to the asymmetric and symmetric C–H vibrations from the F127 [29]. As observed, all these bands corresponding to the carbonyl groups are still present until 873 K, whereas at 1073 K they are completely eliminated, indicating that beyond this temperature only oxidized groups can be expected. Finally, the bands occurring at around 580 and 490 cm^−1^ and observed from 873 K are attributed to the Lu–O stretching vibrations of cubic Lu_2_O_3_ [30], indicating that its crystallization was just beginning at this annealing temperature, as confirmed by XRD. 

**Figure 2:**
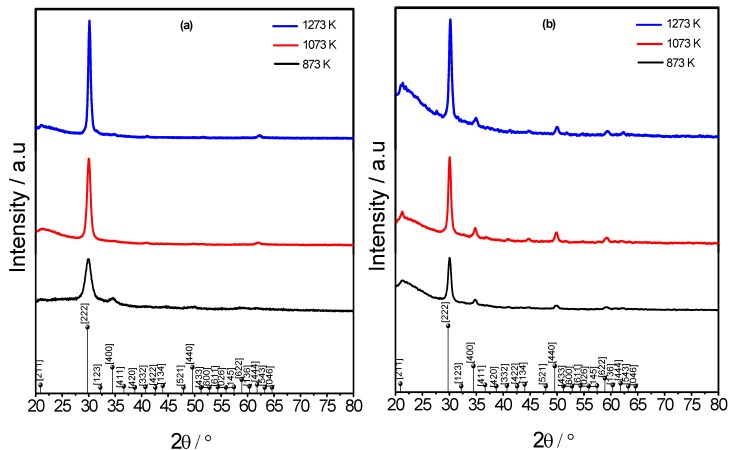
Structural evolution of Lu_2_O_3_:Eu^3+^ F127 modified thin films. (**a**) F127/Lu=1.0; (**b**) F127/Lu = 2.0.

X-ray diffraction patterns of Lu_2_O_3_:Eu^3+^ F127 modified thin films after heat treatment are presented in Figure 2a,b for F127/Lu = 1.0 and 2.0 respectively. The results show that the crystallization only begins when the annealing temperature reaches 873 K, which confirms the results of the IR spectra. At 1073 K the process has formed thin films, crystallized in a cubic bixbyte structure with a spatial group Iā3 [31], and a lattice parameter of 10.391 Å (431021 JCPDS). For films annealed at a higher temperature, more intense and sharp diffraction peaks of Lu_2_O_3_ appear because as the annealing temperature increases the crystallinity of the thin films is enhanced, as can be deduced by the sharpening of the diffraction lines. The crystallite size D of the films can be estimated by Scherrer’s equation and is presented in Table 1. As can be observed, the size ranges from 9.0 to 13.0 nm, and from 14.0 to 17.0 nm for the F127/Lu 1.0 and 2.0 respectively. On the other hand, the films present a preferred <111> orientation. The preferential orientation parameter *α*_hkl_, is defined as *α*_hkl_ *=* *I*_hkl_/*ΣI*_hkl_**, where *I*_hkl_ is the relative intensity of the corresponding diffraction peak [22,32]. As can be seen in Table 1, the calculated preferential orientation of the Lu_2_O_3_:Eu^3+^ F127 modified films increases with the annealing temperature and reaches 97.4% and 84.1% at 1373 K. This behavior is due to the fact that the <111> direction is the lowest surface energy orientation [33,34]. 

**Table 1:** Crystallite size and preferential orientation as a function of the heat treatment temperature of Lu_2_O_3_:Eu^3+^ F127 modified thin films.

Temperature (K)	F127/Lu = 1.0		F127/Lu = 2.0	
	Crystallite size (nm)	<111> orientation	Crystallite size (nm)	<111> orientation
873	9.0	84.1	14.0	79.9
1073	12.0	95.1	15.0	81.3
1273	16.0	97.4	17.0	84.1

**Figure 3:**
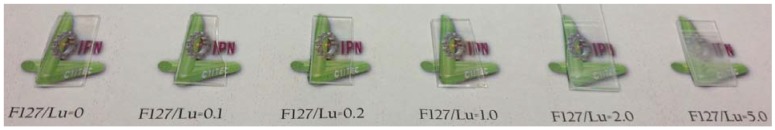
Photograph of transparent Lu_2_O_3_:Eu^3+^ F127 modified thin films.

**Figure 4:**
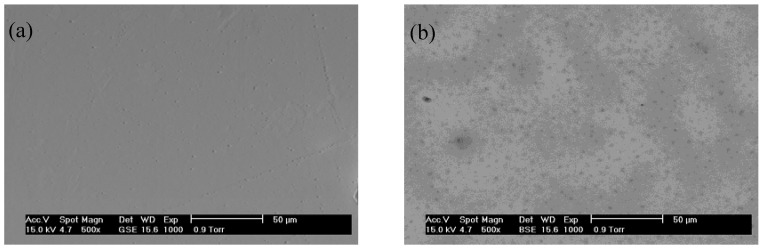
Scanning electron microscope (SEM) micrograph of Lu_2_O_3_:Eu^3+^ F127 modified thin films annealed at 1073 K. (**a**) F127/Lu = 1.0; (**b**) F127/Lu = 2.0.

**Figure 5:**
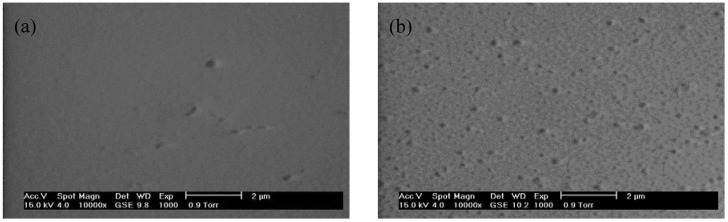
SEM micrograph of the pore content at Lu_2_O_3_:Eu^3+^ F127 modified thin films. (**a**) F127/Lu = 1.0; (**b**) F127/Lu = 2.0.

For luminescent devices, the formation of cracks and pores in the films promotes the likelihood that light will scatter on the film, and therefore the spatial resolution of the formed image will be diminished [15], so it is important to ensure that the films are physically homogenous and transparent. As can be observed in Figure 3, the Lu_2_O_3_:Eu^3+^ modified F127 thin films are completely transparent for F127/Lu from 0 to 2.0. Figure 4a,b present SEM micrographs of a selected area in the F127/Lu 1.0 and 2.0 samples respectively, annealed at 1073 K. With the 1.0 sample, it is observed that the surface is crack free and with lower pore content, whereas the 2.0 sample presents a high pore content, which will decrease the light yield of the sample. Both results were confirmed by means of m-lines spectroscopy. Figure 5a,b shows a zone of the F127/Lu 1.0 and 2.0 samples respectively, at higher magnification. In the F127/Lu = 1.0 sample, the pore content is low with a random distribution, whereas at the F127/Lu = 2.0 presents a high pore content, with its particular morphology which is consequence of the higher F127 level which commonly produces mesoporous materials. However, with lower F127 contents it is possible to obtain almost homogeneous films.

### 2.2. m-Lines Spectroscopy

**Figure 6:**
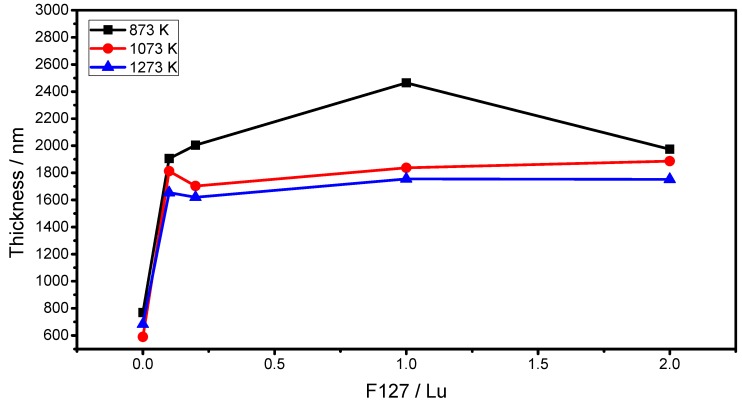
Evolution of thickness of Lu_2_O_3_:Eu^3+^ F127 modified thin films as a function of annealing temperature and F127/Lu molar ratio.

**Figure 7:**
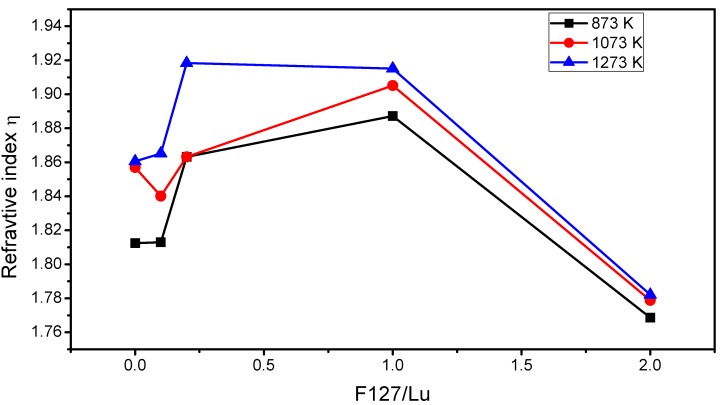
Evolution of refractive index at 633 nm of Lu_2_O_3_:Eu^3+^ F127 modified thin films as a function of annealing temperature and F127/Lu molar ratio.

For luminescent applications, it is crucial that thin films present a high thickness level in order to minimize the effect of the image quality, and m-Lines spectroscopy is a useful method to determine the optogeometric parameters of waveguiding thin films such as refractive index and film thickness [35]. In the present work, after three layers the Lu_2_O_3_:Eu^3+^ modified F127 thin films allow the presence of two TE and two TM modes after different annealing temperatures, from 873 K to 1273 K. The fact that the films could support at least four guided modes demonstrates highly physical homogeneous behavior and high transparency. Evolution of the film thickness and refractive index as the annealing temperature increases in the function of the F127/Lu content are shown in Figure 6 and Figure 7 respectively. 

As expected, with the increment of the annealing temperature the thickness of the film decreases due to a densification process and the elimination of pores in the film. As observed, the increment of the F127/Lu content increments in the thickness of the thin film from 683 to 1653, 1619, 1752, and 1886 nm, and at 1273 K for F127/Lu = 0, 0.1, 0.2, 1.0 and 2.0 respectively, demonstrates that, effectively, the co-polymer acts as a thickness enhancer since the final thickness is 2.7 times higher from the F127/Lu = 0 to the 2.0 sample. However, the increment from the lower F127/Lu content of 0.1 to the higher content of 2.0 only increases the thickness 1.16 times. On the other hand, the refractive index for the same samples rises from 1.86 to 1.92 from the lower F127/Lu content 0.1 to 1.0, and there is a further decrease to 1.78 at the 2.0 sample. This can be explained by the fact that with a higher F127 concentration the film presents higher pore content, as observed in SEM micrographs. However, with lower F127 levels, F127 not only increments the thickness, it also promotes a better crystallization process since the refractive index increases. For the F127/Lu=0.2 and 1.0 sample, the refractive index is close to the theorical for the bulk crystal (1.93). On the other hand, the density of the films can be determined by means of the Lorenz-Lorentz Equation ρ = K (n_f_^2^ − 1)/(n_f_^2^ + 2)^−1^ [36], where n_f_ is the refractive index of the film and K is calculated from the bulk material. The pore content can be calculated by using Drude’s equation (1 − p) = (n_f_^2^ − 1)/(n_b_^2^ − 1)^−1^ where n_b_ is the refractive index of the bulk material, and p is the pore content [37]. The results are presented in Table 2. As shown, the densification ranges from 8.91 to 8.95, 9.36, 9.31, and 8.31 g cm^−3^, at 1273 K for F127/Lu= 0, 0.1, 0.2, 1.0 and 2.0, demonstrating that the obtained thin films are dense and that the F127 also promotes a better densification process at lower F127 contents. Additionally, the pore content decreases from 9.7% to 2.14%, from the lower F127/Lu content of 0.1, which is followed by a substantial increment at the 2.0 sample to 20.18%.

**Table 2:** Density and porosity of Lu_2_O_3_:Eu^3+^ F127 modified thin films as a function of annealing temperature and F127/Lu molar ratio.

Temperature (K)	F127/Lu=0		F127/Lu=0.1		F127/Lu=0.2		F127/Lu=1.0		F127/Lu=2.0	
	Density (g cm^−3^)	Porosity (%)	Density (g cm^−3^)	Porosity (%)	Density (g cm^−3^)	Porosity (%)	Density (g cm^−3^)	Porosity (%)	Density (g cm^−3^)	Porosity (%)
873	8.56	16.1	8.60	15.2	8.93	9.3	9.11	6.03	8.21	21.94
1073	8.89	10.2	8.76	12.4	9.33	1.0	9.24	3.54	8.29	20.61
1273	8.91	9.7	8.95	9.0	9.36	1.6	9.31	2.14	8.31	20.18

### 2.3. Luminescent Properties

**Figure 8:**
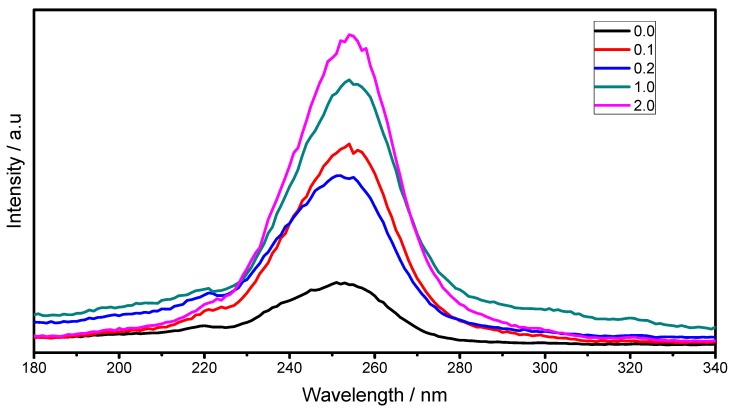
Excitation spectra (λ_em_ = 612 nm) of Lu_2_O_3_:Eu^3+^ F127 modified thin films as a function of F127/Lu molar ratio at annealing temperature of 1073 K.

Figure 8 shows the excitation spectra (λ_em_ = 612 nm) of prepared thin films at different F127/Lu molar ratio (0–2.0), annealed at 1073 K. The broad excitation band from 220 to 280 nm is ascribed to the charge transfer (CT) from the 2p orbital of O^2−^ to the 4f orbital of Eu^3+^ ions. As a consequence of the increment of the F127 content and therefore the thickness, this absorption band increases. 

**Figure 9:**
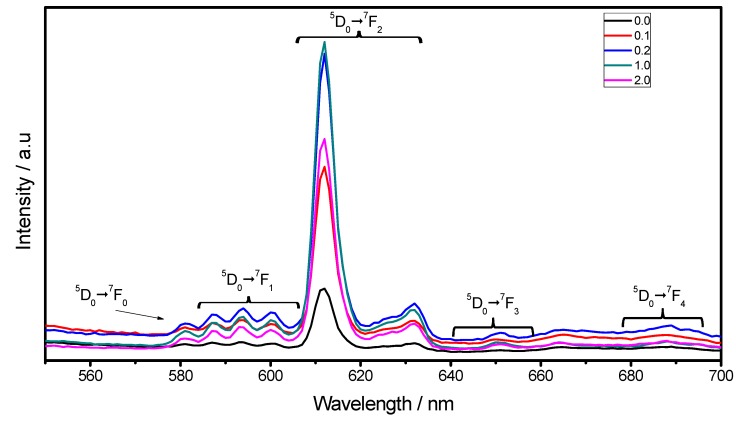
Emission spectra (λ_exc_ = 254 nm) of Lu_2_O_3_:Eu^3+^ F127 modified thin films as a function of F127/Lu molar ratio at annealing temperature of 1073 K.

Almost the same behavior is observed in the emission spectra in Figure 9 (λ_exc_ = 254 nm), where the ^5^D_0_→^7^F_j_ (J = 0, 1, 2, 3, 4) Eu^3+^ transitions can be observed. As observed, for the 2.0 sample a decrement of the luminescence intensity is observed due to the high pore content, which tends to scatter the emission light. The most intense line, at around 612 nm, is ascribed to the transition from the ^5^D_0_→^7^F_2_ of the Eu^3+^. The peak centered at 581 is assigned to the ^5^D_0_→^7^F_0_, transition, the band split in three peaks centered at 588, 594 and 600 nm is ascribed to the ^5^D_0_→^7^F_1_ , whereas the ones centered at 650 and 690 nm are ascribed, to the ^5^D_0_→^7^F_3_ (≈630 nm) and ^5^D_0_→^7^F_4_ transitions. The dominance of the ^5^D_0_→^7^F_2_ electric dipole transition over the ^5^D_0_→^7^F_1_ magnetic dipole transition confirms that the Eu^3+^ ions are preferably located at the non-centrosymetrical C_2_ site in the Lu_2_O_3_ cubic matrix [38]. Therefore the transitions ratio R = I(^5^D_0_→^7^F_2_)/I(^5^D_0_→^7^F_1_) can be used as a reference of the site symmetry [39,40]. The R ratios obtained for F127/Lu = 0, 0.1, 0.2, 1.0 and 2.0 were 3.77 ± 0.06, 4.42 ± 0.04, 5.61 ± 0.07, 7.2 ± 0.05 and 7.2 ± 0.06 respectively, and these results confirm that F127 content is proportional to the site symmetry decreasing. 

**Figure 10:**
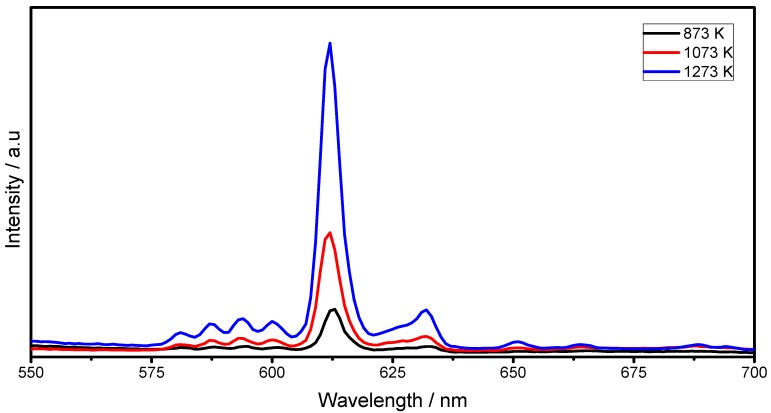
Emission spectra (λ_exc_ = 254 nm) of Lu_2_O_3_:Eu^3+^ F127, with a fixed F127/Lu = 1.0 level as function of annealing temperature.

Finally, Figure 10 shows the effect of the annealing temperature (873–1273 K) for the F127/Lu = 1.0 sample. As observed, the emission increases with the increment of the heat treatment temperature. This behavior can be explained, since as the temperature rises a better crystallization process occurs, and furthermore, as was demonstrated earlier, the films present a higher refractive index and density and lower pore content which contributes to decreasing the scatter of the emissions and therefore increases the signal.

## 3. Experimental Section 

Lu_2_O_3_:Eu^3+^ F127 modified thin films were synthetized using the sol-gel and dip-coating process which is based on the hydrolysis of an inorganic precursor in alcoholic solutions in the presence of an acid catalyst [41]. Firstly, the dissolution of lutetium nitrate (Lu(NO_3_)_3_, Alfa Aesar, 99.9%) in an ethanol-ethylene glycol (C_2_H_6_O–C_2_H_6_O_2_, Fermont, 99.5% and 99.96%, respectively) solution (4:1) was carried out under vigorous stirring at 333 K (60 °C) in order to obtain a sol with a 0.15 M lutetium concentration. Europium nitrate Eu(NO_3_)_3_ (99.5%, Alfa Aesar) was incorporated in order to obtain 2.5 mol % Eu^3+^ samples. The pH solution was adjusted by incorporating acetic acid, C_2_H_4_O_2_ (Fermont 98%), and stabilized with 2,4 pentanedione C_4_H_8_O_3_ (Aldrich, 99%). Finally, F127 was slowly incorporated into the sol, with F127/Lu molar ratio of 0, 0.1, 0.2, 1.0, 2.0 and 5.0 (F127 monomer atomic weight = 102 g mol^−1^). The final sol was stirred for 5 hours at 333 K (60 °C) in order to obtain a complete dissolution of the F127, and it is stable for one day. For the dip-coating procedure, the sols were filtered using a 0.2 µm filter and dipped into the lutetium modified sol with F127 and pulled up at a constant rate of 2 cm s^−1^ on silica glass substrates (refractive index = 1.417) which were carefully cleaned through a special procedure [34]. After each dipping, the films were heat treated in the following cycle: first, dried for 15 min at 373 K (100 °C) in a conventional oven in order to remove the water content and the most volatile solvents; then heat treated at 573 K (300 °C) for 15 min and at 873 K (600 °C) for 15 min in order to achieve a complete removal of the organic residues and promote the densification process without any loss of the physical properties which would diminish the material’s light yield. The dipping cycle was repeated 3 times. Finally, the thin film layers were annealed at a fixed temperature, 1073 K (800 °C) and 1273 K (1000 °C) for 4 hours in order to obtain the desired cubic structure. Most of the produced thin films were completely transparent (Figure 3).

In order to determine the behavior of the sol during the heat treatment, IR spectra were recorded in the 4000–450 cm^−1^ range using Fourier transform infrared spectroscopy (FTIR 2000, Perkin Elmer, 2.0 cm^−1^ resolution). The structure was determined by a D2 Phaser-Bruker diffractometer using a cooper anticathode at 40 kV and 20 mA. The films’ morphology was studied in a Philips XL-30 scanning electron microscope operated at 15 kV. The optogeometrical properties (thickness and refractive index), density and porosity were investigated by m-lines spectroscopy, which uses a prism coupling method to launch a laser light into the optical layer. In optical planar waveguides, light propagation can occur within a layer of a transparent material when its refractive index is higher than that of surrounding layers and when the film has sufficient thickness to support at least one guided mode: either a transverse electric mode, TE_0_, or a magnetic mode, TM_0_. The prism (LaSF35, angle 60°) was coupled with a light of He–Ne laser with a wavelength λ = 633 nm into the waveguide. Finally, the luminescent properties were recorded by employing a Spectra Pro fluorometer equipped with an R955 photomultiplier tube (Hamamatsu), at room temperature.

## 4. Conclusions

The present work synthetized Lu_2_O_3_:Eu^3+^ F127 modified thin films by using a sol-gel process. The results show that transparent, perfectly crystallized, and highly densified Lu_2_O_3_:Eu^3+^ films can be obtained with a F127/Lu molar ratio lower than 1.0. The films crystallize completely at 873 K and are highly orientated at the <111> direction, with an average crystallite size which ranges from 9.2 to 17.1 nm. It was demonstrated that the increment of the F127 content enhances the thickness of the film by approximately 2.7 times compared to a non-modified film. Furthermore, the F127 also promotes better crystallization since the refractive index increases with the increment of the F127 content; and a better densification process and decrement of the pore content are observed. Luminescence studies showed a strong Eu^3+^ 611 nm emission that increases with the F127 content due to the higher thickness and density of the films. The light yield is also increased with the annealing temperature as result of a better crystallization and densification process. The results make the thin films candidates for luminescent applications.
